# Comparison of 5 × 5 Gy and 10 × 3 Gy for metastatic spinal cord compression using data from three prospective trials

**DOI:** 10.1186/s13014-020-01737-7

**Published:** 2021-01-07

**Authors:** Dirk Rades, Jon Cacicedo, Antonio J. Conde-Moreno, Barbara Segedin, Darejan Lomidze, Raquel Ciervide, Niels H. Hollaender, Steven E. Schild

**Affiliations:** 1 Department of Radiation Oncology, University of Lübeck, Ratzeburger Allee 160, 23562 Lübeck, Germany; 2 Department of Radiation Oncology, Cruces University Hospital/Biocruces Health Research Institute, Barakaldo, Spain; 3 Department of Radiation Oncology, University and Polytechnic Hospital La Fe, Valencia, Spain; 4 Department of Radiotherapy, Institute of Oncology Ljubljana, Ljubljana, Slovenia; 5 Radiation Oncology Department, HTMC University Clinic Tbilisi, Tbilisi, Georgia; 6 Department of Radiation Oncology, University Hospital HM Hospitales, Sanchinarro, Madrid, Spain; 7 Department of Oncology and Palliative Units, Zealand University Hospital, Naestved, Denmark; 8 Department of Radiation Oncology, Mayo Clinic, Scottsdale, AZ USA

**Keywords:** Metastatic spinal cord compression, Radiotherapy alone, Local progression-free survival, Motor function, Ambulatory status, Overall survival

## Abstract

**Background:**

In a palliative situation like metastatic spinal cord compression (MSCC), overall treatment time of radiotherapy should be as short as possible. This study compared 5 × 5 Gy in 1 week to 10 × 3 Gy in 2 weeks in a prospective cohort.

**Methods:**

Forty patients receiving 5 × 5 Gy in a phase II trial were matched 1:2 to 213 patients receiving 10 × 3 Gy in two previous prospective studies for tumor type, ambulatory status, time developing motor deficits, interval between tumor diagnosis and MSCC and visceral metastases. These factors were consistent in all three patients (triple) used for each 1:2 matching. Groups were compared for local progression-free survival (LPFS), motor function, ambulatory status, and overall survival (OS).

**Results:**

After matching, 32 triples remained for analyses (N = 96 in total). Six-month LPFS-rates were 94% after 5 × 5 Gy and 87% after 10 × 3 Gy (*p* = 0.36), 6-month OS-rates 43% and 35% (*p* = 0.74). Improvement of motor function was achieved in 59% and 34% of patients (*p* = 0.028); overall response rates (improvement or no further progression of motor deficits) were 94% and 89% (*p* = 0.71). Post-treatment ambulatory rates were 81% after 5 × 5 Gy and 85% after 10 × 3 Gy (*p* = 0.61). Of non-ambulatory patients, 50% (6/12) and 46% (11/24) regained the ability to walk (*p* = 1.00).

**Conclusions:**

5 × 5 Gy in 1 week appeared similarly effective as 10 × 3 Gy in 2 weeks. These results may not be applicable to long-term survivors and should be confirmed in a randomized trial directly comparing 5 × 5 Gy and 10 × 3 Gy.

*Trial registration* clinicaltrials.gov NCT03070431. Registered 27 February 2017.

## Background

Metastatic spinal cord compression (MSCC) occurs in 5–10% of patients with malignant diseases [1–3]. Many of these patients receive radiotherapy alone. Different dose-fractionation regimens are available [1–3]. One common regimen is 10 × 3 Gy in 2 weeks. According to previous studies, such longer-course programs are not superior to short-course programs regarding motor function but can lead to improved local progression-free survival (LPFS) [4–6]. This may be explained by the higher equivalent dose in 2 Gy-fractions (EQD2) of 10 × 3 Gy (32.5 Gy) for tumor cell kill compared to 5 × 4 Gy (23.3 Gy) [7]. Since treatment sessions can be inconvenient for these patients, the ideal radiation program should be effective and short. These criteria may be met by 5 × 5 Gy in 1 week with an EQD2 similar to 10 × 3 Gy. In a previous phase II trial, 5 × 5 Gy resulted in significantly better LPFS than 5 × 4 Gy [8]. A comparison between 5 × 5 Gy and 10 × 3 Gy was lacking, so we performed this analysis using data of three prospective studies [8–10].

## Patients and methods

Patients in the phase II trial (PRE-MODE, clinicaltrials.gov: NCT03070431) received precision radiotherapy with 5 × 5 Gy in 1 week between 02/2017 and 03/2018 [8]. The current study (secondary analysis) received approval from the ethics committee (University of Lübeck, 16–163) in August 2020. Details of the PRE-MODE trial were previously reported [8]. Its primary endpoint was 6-month LPFS, defined as lack of progressive motor deficits during radiotherapy and freedom from in-field recurrence of MSCC thereafter. Secondary objectives included effect on motor function, post-treatment ambulatory status and overall survival (OS). For motor function, the following grading-system was applied: 0 = normal strength; 1 = ambulatory without aid; 2 = ambulatory with aid; 3 = not ambulatory; 4 = paraplegia [11]. Improvement or deterioration was defined as change of ≥ 1 point.

Since the EQD2 of 5 × 5 Gy (31.3 Gy) for tumor cell kill (alpha/beta ratio 10 Gy) is similar to 10 × 3 Gy (32.5 Gy), it is assumed that both regimens are similarly effective [7]. Patients of the PRE-MODE trial (5 × 5 Gy) were compared to patients receiving 10 × 3 Gy in a prospective non-randomized study (SCORE-1, 01/06–12/07) or a phase III trial (SCORE-2, 07/10-05/15; clinicaltrials.gov: NCT02189473) [9, 10]. To avoid a potential bias due to follow-up time, follow-up in the 10 × 3 Gy group was censored at 6 months.

The 40 patients of the PRE-MODE trial were matched 1:2 to the 213 patients receiving 10 × 3 Gy in a previous trial [9, 10]. Matching criteria included primary tumor type (breast cancer vs. prostate cancer vs. myeloma/lymphoma vs. lung cancer vs. others), pre-treatment ambulatory status (not ambulatory vs. ambulatory), time developing motor deficits prior to radiotherapy (faster, 1–7 days vs. slower, > 7 days), interval between tumor diagnosis and MSCC (≤ 12 vs. > 12 months), and visceral metastases (no vs. yes). Four criteria were identified in prospective studies as significantly associated with motor function and ambulatory status [12–14]. Visceral metastasis was identified as negative predictor of local control and LPFS [5, 6]. These five factors were consistent in all three patients (triple) used for each 1:2 matching. Groups were compared for LPFS, effect on motor function (improvement, overall response), post-treatment ambulatory status and OS. In addition, median age, gender, Eastern Cooperative Oncology Group performance score (ECOG 1–2 vs. 3–4), number of affected vertebrae (1–2 vs. ≥ 3) and additional bone metastases (no vs. yes) were compared.

Comparisons for LPFS and OS were performed using Kaplan–Meier method and log-rank test. For the comparison regarding age, the Mann-Whiney U test was used. The comparisons for patient characteristics, improvement of motor function, overall response and ambulatory status were performed with the Fisher’s exact test.

## Results

After 1:2 matching, 32 triples remained for analyses, corresponding to a total of 96 patients. The distribution of the patient characteristics was not significantly different (Table 1). When applying the Mann-Whiney U test for comparison of median age, the *z-*score was 0.933, and distribution was considered approximately normal.

**Table 1:** Distribution of matching criteria and other patient characteristics in the cohort of patients receiving 5 × 5 Gy (n = 32) and in those patients receiving 10 × 3 Gy (n = 64)

	5 × 5 Gy N patients	10 × 3 Gy N patients	*P*
Type of primary tumor			1.00
Breast cancer	7 (22%)	14 (22%)	
Prostate cancer	2 (6%)	4 (6%)	
Myeloma/lymphoma	3 (9%)	6 (9%)	
Lung cancer	9 (28%)	18 (28%)	
Other tumors	11 (34%)	22 (34%)	
Ambulatory prior to radiotherapy			1.00
Not ambulatory	12 (38%)	24 (38%)	
Ambulatory	20 (63%)	40 (63%)	
Time developing motor deficits prior to radiotherapy			1.00
Faster development (1–7 days)	9 (28%)	18 (28%)	
Slower development (> 7 days)	23 (72%)	46 (72%)	
Interval between tumor diagnosis and MSCC			1.00
≤ 12 months	17 (53%)	34 (53%)	
> 12 months	15 (47%)	39 (47%)	
Visceral metastases at the time of radiotherapy			1.00
No	11 (34%)	22 (34%)	
Yes	21 (66%)	42 (66%)	
Median age (range)	63 (36–79) years	65 (38–86) years	0.35
Gender			0.83
Female	13 (41%)	28 (44%)	
Male	19 (59%)	36 (56%)	
Eastern Cooperative Oncology Group performance score			0.66
1–2	15 (47%)	26 (41%)	
3–4	17 (53%)	38 (59%)	
Number of vertebrae affected by MESCC			0.83
1–2	20 (63%)	38 (59%)	
3	12 (38%)	26 (41%)	
Other bone metastases at the time of radiotherapy			1.00
No	7 (22%)	15 (23%)	
Yes	25 (78%)	49 (77%)	

The *p* value for the comparison regarding age was obtained with the Mann-Whiney U test, otherwise *p* values were obtained with the Fisher’s exact test

**Fig. 1:**
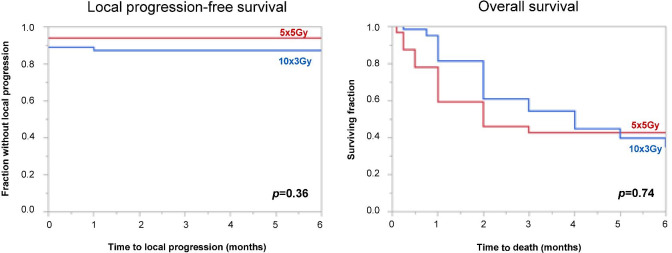
Kaplan–Meier curves of the comparisons between 5 × 5 Gy and 10 × 3 Gy for local progression-free survival (left) and overall survival (right). The *p* values were calculated using the log-rank test

Six-month LPFS-rates were 94% after 5 × 5 Gy and 87% after 10 × 3 Gy (Fig. 1, *p* = 0.36), and 6-month OS-rates 43% and 35% (Fig. 1, *p* = 0.74). Improvement of motor function was achieved in 59% (19/32) and 34% (22/64) of patients (*p* = 0.028). Overall response rates were 94% (30/32) and 89% (57/64) (*p* = 0.71). Deterioration of motor deficits occurred in 6% (2/32) and 11% (7/64) of patients (*p* = 0.71). Post-treatment ambulatory rates were 81% after 5 × 5 Gy and 85% after 10 × 3 Gy (*p* = 0.61). Of non-ambulatory patients, 50% (6/12) after 5 × 5 Gy and 46% (11/24) after 10 × 3 Gy, respectively, regained walking ability (*p* = 1.00). Of ambulatory patients, 100% (20/20) and 93% (37/40), respectively, remained ambulatory (*p* = 0.54).

## Discussion

Important endpoints of radiotherapy for MSCC include functional outcome and local control [1–3]. In several studies, short-course and longer-course regimens were similarly effective for functional outcome [4–6]. Local control of MSCC was better after longer-course radiotherapy such as 10 × 3 Gy, most likely due to higher EQD2 [5, 6]. In a phase II trial, 5 × 5 Gy in 1 week resulted in significantly better LPFS (*p* = 0.026) when compared to a historical control group receiving 5 × 4 Gy [8]. EQD2 for tumor cell kill of 5 × 5 Gy is similar to 10 × 3 Gy [8]. 5 × 5 Gy could become important for MSCC, if it is as effective as 10 × 3 Gy, since treatment time could be reduced from 2 weeks to 1 week.

This study compared 5 × 5 Gy to 10 × 3 Gy using data of three prospective studies. Forty patients receiving 5 × 5 Gy were matched 1:2 to 213 patients receiving 10 × 3 Gy. Matching criteria were selected according to previous studies [5, 6, 12–14]. Triples were matched for primary tumor type, pre-treatment ambulatory status and visceral metastases according to the phase II trial [8]. For the time developing motor deficits prior to radiotherapy, two instead of three categories were used. In accordance with the larger prospective study, 1–7 and > 7 days were selected [9]. After applying the four criteria, 33 triples remained. In the 99 patients, the median interval between tumor diagnosis and MSCC was 12 months. Therefore, ≤ 12 and > 12 months were selected for the matching. After application of all five criteria, 32 triples (96 patients) remained.

5 × 5 Gy resulted in a significantly higher rate of improvement of motor function than 10 × 3 Gy. For the other investigated endpoints including the main objective LPFS, no significant differences were found. Thus, 5 × 5 Gy appeared similarly effective as 10 × 3 Gy. When interpreting these results, the limitations of the study must be considered including the non-randomized design. To minimize the risk of hidden selection biases, we used only data from prospective studies. Further limitations include the facts that follow-up MRI was not performed at pre-defined time points and that for grading of MSCC radiological criteria were not considered [8–10, 15]. Furthermore, the trials were performed during different time periods. Systemic treatment for metastatic cancer has improved over time, particularly since the introduction of new targeted therapies such as immune checkpoint inhibitors [16]. The fact that more than half of the patients died within 6 months following radiotherapy demonstrates that the situation was often absolutely palliative. Therefore, present findings may not be transferred to long-term survivors. Patients with favorable survival prognoses can benefit from radiotherapy with higher doses or upfront surgery [17, 18]. When aiming to deliver higher doses, stereotactic body radiation therapy (SBRT) is an option, which has been successfully administered for painful spinal metastases [19]. SBRT is usually recommended for patients with favorable survival prognoses and 1–3 spinal metastases, if not more than two contiguous vertebrae are affected by MSCC. This applied also to the majority of patients in this study. When using 5 × 5 Gy, not all dose constraints for spinal irradiation with 5 fractions may be met if more than two contiguous vertebrae are involved and spinal MRI is not available, and treatment outcomes may be less favorable [20–23].

## Conclusions

5 × 5 Gy in 1 week appeared similarly effective as 10 × 3 Gy in 2 weeks for LPFS, functional outcome and OS. These results may not be applicable to long-term survivors and should be confirmed in a randomized trial that directly compares 5 × 5 Gy and 10 × 3 Gy.

## Data Availability

Data of the PRE-MODE trial (NCT03070431) and the SCORE-2 trial (NCT02189473) are available at clinicaltrials.gov.

## Abbreviations

ECOG: Eastern Cooperative Oncology Group
EQD2: Equivalent dose in 2 Gy fractions
LPFS: Local progression-free survival
MSCC: Metastatic spinal cord compression
OS: Overall survival
SBRT: Stereotactic body radiation therapy
